# Generating rate equations for complex enzyme systems by a computer-assisted systematic method

**DOI:** 10.1186/1471-2105-10-238

**Published:** 2009-08-04

**Authors:** Feng Qi, Ranjan K Dash, Yu Han, Daniel A Beard

**Affiliations:** 1 Biotechnology and Bioengineering Center and Department of Physiology, Medical College of Wisconsin, Milwaukee, Wisconsin, 53226, USA

## Abstract

**Background:**

While the theory of enzyme kinetics is fundamental to analyzing and simulating biochemical systems, the derivation of rate equations for complex mechanisms for enzyme-catalyzed reactions is cumbersome and error prone. Therefore, a number of algorithms and related computer programs have been developed to assist in such derivations. Yet although a number of algorithms, programs, and software packages are reported in the literature, one or more significant limitation is associated with each of these tools. Furthermore, none is freely available for download and use by the community.

**Results:**

We have implemented an algorithm based on the schematic method of King and Altman (KA) that employs the topological theory of linear graphs for systematic generation of valid reaction patterns in a GUI-based stand-alone computer program called *KAPattern*. The underlying algorithm allows for the assumption steady-state, rapid equilibrium-binding, and/or irreversibility for individual steps in catalytic mechanisms. The program can automatically generate MathML and MATLAB output files that users can easily incorporate into simulation programs.

**Conclusion:**

A computer program, called *KAPattern*, for generating rate equations for complex enzyme system is a freely available and can be accessed at .

## Background

Since Haldane's analysis of a simple enzyme mechanism [1], kinetic analysis has been central to our quantitative understanding of enzyme mechanisms [2,3]. In conventional applications, kinetic data from initial-rate experiments are used to evaluate enzyme mechanisms based upon derived mechanistic rate expressions. Such rate expressions are important in building integrated models of metabolic systems which involve a number of enzymatic reactions [4,5]. In principle, the rate equations for a given discrete-state reaction mechanism can be derived by solving a system of simultaneous nonlinear algebraic equations that result from the steady-state expressions for the concentrations of all of the enzyme intermediates. This approach was first applied successfully by Botts and Morales [6] to some enzymatic systems. However, when the system involves multiple substrates, enzyme complexes, and products [1], deriving rate equations based on steady-state equations may be too complex to be of practical interest and also can be liable to human errors. Therefore, systematic approaches, as reviewed by Huang [7], are desirable.

King and Altman [8] introduced a graphical/schematic method for facilitating derivation of steady-state rate equations in enzymatic systems. Modifications introduced by Volkenstein and Goldsein [9] and Cha [10] added substantial power to the King-Altman method by applying graph theory and allowing for the assumption that one or more of the reversible steps in the enzyme mechanism is maintained in rapid equilibrium [10]. Other alternative methods include those described by Fromm [11], Orsi [12], Ainsworth [13,14], Indge and Childs [15], and Chou and Forsen [16].

Even when using graphical methods, manually deriving the steady-state rate equations for non-trivial enzyme mechanisms can be cumbersome and error-prone. Therefore, computer-assisted methods are useful. Applying the method of King-Altman, Pring [17] and Rhoads [18] developed two programmes, *K *and *D*, which perform logical operations essential for generating rate equations based on the strictly steady-state assumption with respect to a certain class of species present. Lam and Priest [19] introduced an algorithm based on graph theory that is computer programmable. Cornish-Bowden [20] presented a computer implementation of Cha's method using an exhaustive search. A computer program developed by Kinderlerer and Ainsworth [21] is restricted to enzyme mechanisms involving up to 10 enzyme intermediates. Straathof and Heijnen [22] and Fromm and Fromm [23] introduced methods to derive rate equations for enzyme systems using the symbolic algebra packages Maple and Mathematica. However, these programs derive only strictly steady-state rate equations and cannot obtain rate equations involving irreversible steps. Varon *et al*. [24] developed a program called Albass that overcame many of the limitations of earlier programs. Several years later, the Varon group developed two new programs written in C++, called Referass [25] and WinStes [26], which can derive rate equations for mechanisms with up to 255 intermediate states with up to 255 reactions. The algorithms and software developed by Varon and colleagues represent the most powerful and flexible previously developed tools for deriving enzyme rate expressions. Yet, like other previous packages, it does not appear to be currently available.

We present here a simple, stand-alone computer program written in MATLAB GUI, called *KAPattern*, for generating rate equations in complex enzyme systems. This program is based on the schematic method of King and Altman [8], and uses the topological theory of linear graphs, called Wang Algebra [27], that systematically generates valid King-Altman directed graph patterns. Our package provides the functionality of the WinStes program of Varon and colleagues (in that it can handle strictly steady-state as well as quasi-equilibrium steps, can be applied to branched as well as unbranched systems, and does not rely on an exhaustive search for determining directed graphs) with several additional features:

1. There is no limitation on the size of the system other than that imposed by the available memory and CPU resources.

2. The program can output the results (the generated rate equations) as a MathML file or a MATLAB .*m *file which may be integrated into simulation program. (For instance, it can be used in conjunction with a simulation package such as *BISEN *[28].)

3. The program provides visualization of all the valid KA patterns.

4. Functions available in *KAPattern *may help the end-users to obtain insights on catalytic mechanism (e.g., structural properties, topological features, stoichiometric matrix etc.) that may be useful for other applications.

5. Foremost, the package is freely available for download and use by the community.

## Results and Discussion

### The King-Altman method

**Figure 1:**
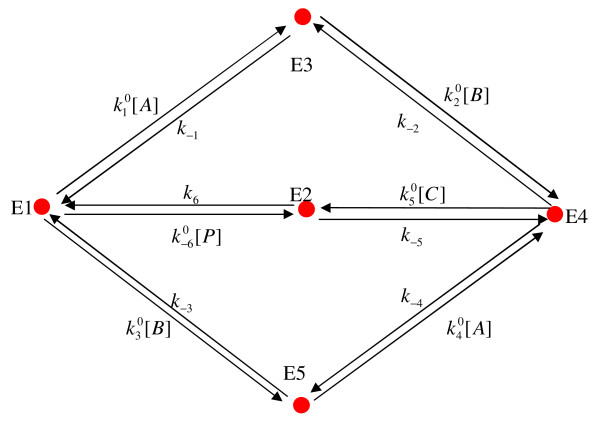
**Illustration of an enzyme-catalyzed reaction mechanism: fumarase**. Here *E*_*i *_is *i*th enzyme form, *A *is fumarate (substrate), *B *is proton, *C *is hydroxyl, and *P *is malate (product). This mechanism is proposed in [29].

The King-Altman (KA) procedure is easily understood based on an illustrative example, as described here for the enzyme mechanism illustrated in Figure 1. This mechanism is the proposed five-state catalytic scheme for fumarase (or fumarate hydratase), which catalyzes the hydration of fumarate to malate [29]. This mechanism involves 5 enzyme states (*n *= 5) and 6 links between those states, characterized by 12 rate constants.

**Figure 2:**
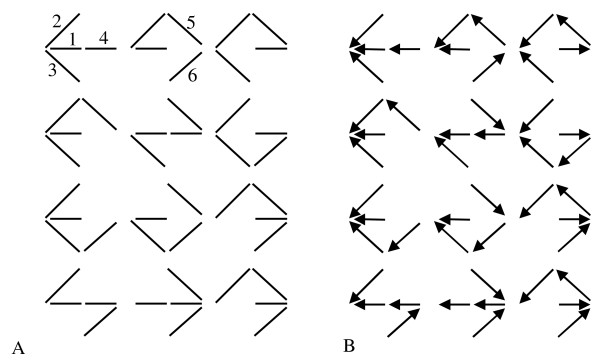
**The graph representation of the valid reaction patterns generated by the King-Altman (KA) method**. (A) Valid KA patterns. (B) Directional diagrams associated with the enzyme state 1. The numbers in plot A are the indexes of the links in the linear graph representation of the mechanism. Using the cut matrix method introduced in the text, one can easily get that the number of valid KA patterns is 12. Accordingly, the number of directional diagrams associated with the 5 enzyme states is 5 × 12 = 60.

The first step in applying the KA procedure is listing all of the valid KA patterns for the enzyme mechanism. These patterns, illustrated in Figure 2A, are the set of all subsets of the graph in Figure 1, with the maximum number of edges while excluding any closed loops. There there are 12 possible KA patterns associated with the mechanism of Figure 1.

The next step is to determine all of the *directional diagrams *associated with each state in the enzyme mechanism. The directional diagrams associated with a given state are constructed from the KA pattern set, with directions indicated on each edge on each KA pattern. The directions are chosen so that, for state *i*, the arrows are directed toward state *i *with no diverging edges. The set of all directional diagrams associated with state 1 for the mechanism of Figure 1 is illustrated in Figure 2B. Since there are 5 states and 12 KA patterns, there are 5 × 12 = 60 directional diagrams associated with the mechanism of Figure 1. Each directional diagram is associated with a product of pseudo-first order rate constants for the arrows in the directional diagram. For example, the term for the top-left directional diagram in Figure 2B is . The relative steady-state concentration of each state is proportional to the summation of 12 terms associated with the 12 directional diagrams for each state. Specifically, the relative concentration of the *i*th enzyme state can be computed as a fraction of the total enzyme concentration whose numerator is the sum of the 12 terms associated with the 12 directional diagrams which all point or end to *i*th state (for example, as shown in Figure 2B, 12 directional diagrams point or end to state 1), and denominator is the sum of all 60 terms associated with the directional diagrams for all 5 states in the system (5 × 12). That can be written as:

(1)

Here Σ_*i *_represents the sum of the 12 terms associated with the state *i *and Σ is the sum over all 5 sets of 12 terms for all states, and *E*_*o *_is the total enzyme concentration.

The KA method is described in somewhat more detail in [30] and [31].

### Algorithm

As described above, the graphical method of King and Altman is based on determining a set of KA patterns that are subsets of the graph of the enzyme mechanism. Each KA pattern contains the maximal number of edges possible while not containing any closed loops. Each enzyme state (each vertex in the graph) has associated with it a directional diagram for each KA pattern. Enumerating all directional diagrams becomes more difficult as the enzyme mechanism becomes more complex.

Previous applications of the theory of graphs to the solution of enzyme kinetic problems have been aimed at developing algorithms that are easy to program and allow users to rapidly calculate the steady-state concentrations of enzyme states, and thereby obtain expressions for the rate of product accumulation [9,11]. Unlike using symbolic algebra packages to solve a set of nonlinear algebraic equations based on steady-state and mass conservation, these approaches take advantage of the similarity between complex enzyme mechanisms and electrical networks. Specifically, it has been proved that the method used to generate trees from linear graphs can be applied to complex enzymatic reaction mechanisms [32].

Here we use the method described by Lam and Priest [19] to automatically generate the valid KA patterns from the reaction graph. This method makes use of the theory called Wang Algebra [27], where the key principle is that the addition or multiplication operation on two or more identical elements leads to zero (none). This property can be expressed as

(2)

(3)

where *c *is a element which has been operated. We will see below how this property can be applied as an algebraic representation of a requirement for valid KA patterns.

**Figure 3:**
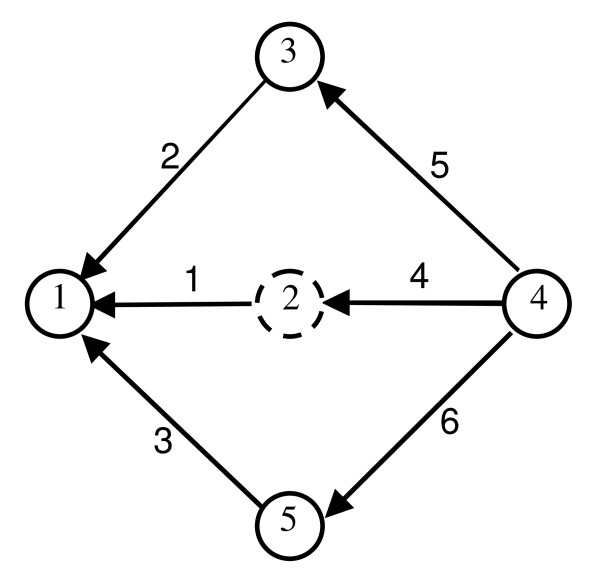
**Linear graph representation of the enzyme catalyzed reaction mechanism of fumarase shown in Figure 1**. The numbers inside the circles are the indexes of enzyme forms in the system. The numbers along the lines which connecting the nodes are indexes of links assigned by the program. Directions are assigned arbitrarily to get the cut matrix , see text for details.

To apply the Lam-Priest algorithm, we first simplify and re-plot the enzymatic system as shown in Figure 3. In this linear graph representation, a node (vertex) represents the enzyme form and a link (edge) represents the inter-conversion relationship between two enzyme forms. That means if there is inter-conversion between two enzyme forms, whether reversible or irreversible, then there is a link to connect them. The links are nondirectional. A number is assigned to each node (vertex), and each link (edge). The linear graph structure is represented by an *n *× *n *symmetrical matrix (called the link matrix ℒ in our program). For the example of Figure 3, ℒ can be written as:

(4)

Each element ℒ_*ij *_is the index of the link between node (enzyme state) *i *and *j*.

Further information on the kinetic mechanism of the reaction system is stored in an *n *× *n *matrix (called kinetic matrix  in our program). For the current example,  is given by:

(5)

Note that  is not symmetric in general.

Application of the Lamb-Priest algorithm starts with randomly selecting *n *- 1 nodes from the linear graph, and determining the links connected to the *n *-1 nodes. (It makes no difference which node is excluded; the same final results are obtained for any arbitrary choices.) It is easy to carry out this operation on ℒ by deleting a row (column) and then listing separately all the nonzero entries from the remaining *n *- 1 rows (columns). In the next step, using the Wang Algebra described above, the links listing obtained in previous step are *alphanumerically *multiplied. Here the alphanumeric multiplication of elements (integers or other symbols) is defined as a list rather than numerical values. For example, multiplying alphanumerically 1 and 2 is equal 12 rather than 2. The result of this operation is the set of all valid KA patterns, expressed by a set of link index array. The Wang Algebra principle guarantees that no invalid or redundant patterns are generated through these steps.

In the final step, the links (edges) in each KA pattern are assigned directions so that the reaction steps, individually or in sequence, lead to a given enzyme state *E*_*i*_. For example, if we delete the second row corresponding to the enzyme form 2 (surround by dashed circle in Figure 3), we obtain E1 (1 2 3); E3 (2 5); E4 (4 5 6); E5 (3 6). In the next step, we alphanumerically multiply these four lists:

(6)

Thus, an alphanumerical multiplication of two lists, such as (1 2 3) and (2 5), yields a list of all of the entry-by-entry products, (1–2 1–5 2–5 2–3 3–5) (Recall that the product of identical elements, such as 2-2 is discarded. The product is discarded if it shows up more than once.). The result lists all the valid KA patterns represented by 12 lists of link indexes. The graph representation of all the valid KA patterns is shown in Figure 2A. The algorithm ensures that only one link shows up in one single pattern only once (Equation (2) and Equation (3)), and that there are no redundant patterns.

As an internal check, we apply an independent method to calculate the expected number of valid KA patterns. Several such methods are available, including one introduced by Chou and Forsen [16] based on the Wong-Hanes rule [33,34]. A simpler method is to calculate the determinant of the product of the cut matrix  and its transpose, yielding the correct number of valid KA patterns for any enzyme mechanism [27]. The cut matrix is defined by using the *n *- 1 nodes of *n *enzyme complexes as the row numbers and the link indexes as the column numbers. Thus

(7)

where

(8)

Here, *n *= total number of enzyme forms and *m *= total number of links. Each entry is defined *c*_*i*,*j *_= 1 if link *j *enters state *i*, *c*_*i*,*j *_= -1 if link *j *leaves enzyme *i*, and = 0 otherwise. Thus there is a different definition of the cut matrix  for each set of definitions of link directions. For the purpose of computing the number of valid KA patterns, the assignment of directions of these links is arbitrary. For example, if we exclude node 2 (dashed circled in Figure 3) and assign directions for each link as shown in Figure 3, the cut matrix will be:

(9)

and



## Implementation

We use the enzyme-catalyzed reaction mechanism of fumarase to illustrate the usage of the *KAPattern *program. Given a simple input file for this complex enzymatic reaction, this program produces the link matrix ℒ and kinetic matrix  as well as generates all the valid KA patterns, outputs each pathway corresponding to each enzyme form based on the generated valid patterns, and outputs the results (i.e., the generated rate equations) as a MathML file or a MATLAB .*m *file, which can be used in a simulation program of an integrated metabolic system model.

In this program, the input file for an enzyme-catalyzed reaction mechanism is a simple .*txt *file that lists every pseudo-first-order rate constants in the enzyme catalytic system. Below is the input file for the fumarase reaction mechanism; the first column and the second column are the indexes of the enzyme forms, the third column is the pseudo-first-order rate constants connecting the corresponding two enzyme forms (transferring from the first to the second):

input.txt

1 3 k1*A

3 1 k_1

1 2 k_6*P

2 1 k6

1 5 k3*B

5 1 k_3

2 4 k_5

4 2 k5*C

3 4 k2*B

4 3 k_2

4 5 k_4

5 4 k4*A

To clarify, the first line of the input.txt file is 1 3 k1*A, which means the rate constant for the enzyme conversion from form 1 (E1) to 3 (E3) is k1*A. The functions ReadInput and GetLink in our *KAPattern *program, read the input file and generate the matrices ℒ (Equation (4)) and  (Equation (5)). The function Wang is used to generate the valid KA patterns. (For detailed description of functions and the full example, see the additional file 1: Appendix.)

With all the valid KA patterns generated, it is straightforward to enumerate all of the directional diagrams using the information from the  and ℒ matrices. For the enzyme form *E*_*i*_, the program checks each non-zero entry of the *i*th column in the ℒ matrix against all links in the link list of one pattern and finds every link that points to the enzyme form *E*_*i*_. Based on the next end point, the process is repeated until no links is left out in the list. Finally, multiplying all the pseudo-first-order rate constants, we can get the expression corresponding to one pattern. Repeating this procedure for each KA pattern and for each enzyme form, we obtain the concentration of each enzyme form *E*_*i *_relative to the total concentration of enzyme *E*_*o*_.

The cost of the rate equation generation depends not only on the size of the problem, but also on the complexity of the problem. For most small-sized enzyme systems we tested, the program gives results less than 1 second. For the moderate-size problem example we present in the additional file (See the additional file 1: Appendix.), the program generates 288 valid KA patterns in about 4.5 seconds (on Intel Pentium IV, 2.0 GHz, 2 GB RAM).

Below is the MATLAB output file generated from the function BuildFile of the *KAPattern *program for the fumarase reaction mechanism. The output has one or two input parameters, depending on whether there are pseudo-first-order rate constants, and three output variables. If there are pseudo-first-order rate constants, which include substrates or products concentrations, the two parameters will be two arrays K and Con, corresponding to reaction rate constants and substrate and product concentrations, respectively. The three output variables are N, F, and D, where N and F are vectors listing the numerators Σ_*i *_and the fractions Σ_*i*_/Σ for Equation (1). The output variable D is the denominator D.

function [E, F, D] = Expression(K, Con)

%% concentration values

A = Con(1);

B = Con(2);

C = Con(3);

P = Con(4);

%% rate constant k values

k1 = K(1);

k2 = K(2);

k3 = K(3);

k4 = K(4);

k5 = K(5);

k6 = K(6);

k_1 = K(7);

k_2 = K(8);

k_3 = K(9);

k_4 = K(10);

k_5 = K(11);

k_6 = K(12);

%% numerators

E(1) = k6*k_1* k_3*k5*C + k6*k_1*k_3*k_2 + k6*k_1*k_3*k_4 + k6*k_1*k5*C*k4*A + k6*k_1*k_2*k4*A + k6*k_3*k5*C*k2*B + k6*k_3*k_4*k2*B + k6*k5*C*k2*B*k4*A + k_1*k_3*k_2*k_5 + k_1*k_3*k_4*k_5 + k_1*k_2*k_5*k4*A + k_3*k_4*k_5*k2*B;

E(2) = k_6*P*k5*C*k_1*k_3 + k_6*P*k_1*k_3*k_2 + k_6*P*k_1*k_3*k_4 + k_6*P*k5*C*k_1*k4*A + k_6*P*k_1*k_2*k4*A + k_6*P*k5*C*k_3*k2*B + k_6*P*k_3*k_4*k2*B + k_6*P*k5*C*k2*B*k4*A + k5*C*k2*B*k1*A*k_3 + k5*C*k4*A*k3*B*k_1 + k5*C*k2*B*k4*A*k1*A + k5*C*k2*B*k4*A*k3*B;

E(3) = k1*A*k6*k_3*k5*C + k1*A*k_2*k6*k_3 + k1*A*k6*k_3*k_4 + k1*A*k6*k5*C*k4*A + k1*A*k_2*k6*k4*A + k_2*k_5*k_6*P*k_3 + k_2*k4*A*k3*B*k6 + k_2*k_5*k4*A*k_6*P + k1*A*k_2*k_3*k_5 + k1*A*k_3*k_4*k_5 + k1*A*k_2*k_5*k4*A + k_2*k_5*k4*A*k3*B;

E(4) = k_5*k_6*P*k_1*k_3 + k2*B*k1*A*k6*k_3 + k4*A*k3*B*k6*k_1 + k_5*k4*A*k_6*P*k_1 + k2*B*k4*A*k1*A*k6 + k_5*k2*B*k_6*P*k_3 + k2*B*k4*A*k3*B*k6 + k_5*k2*B*k4*A*k_6*P + k_5*k2*B*k1*A*k_3 + k_5*k4*A*k3*B*k_1 + k_5*k2*B*k4*A*k1*A + k_5*k2*B*k4*A*k3*B;

E(5) = k3*B*k6*k_1*k5*C + k3*B*k6*k_1*k_2 + k3*B*k_4*k6*k_1 + k_4*k_5*k_6*P*k_1 + k_4*k2*B*k1*A*k6 + k3*B*k6*k5*C*k2*B + k3*B*k_4*k6*k2*B + k_4*k_5*k2*B*k_6*P + k3*B*k_1*k_2*k_5 + k3*B*k_4*k_1*k_5 + k_4*k_5*k2*B*k1*A + k3*B*k_4*k_5*k2*B;

%% denominator

D = E(1) + E(2) + E(3) + E(4) + E(5);

%% fractions

F(1) = E(1)/D;

F(2) = E(2)/D;

F(3) = E(3)/D;

F(4) = E(4)/D;

F(5) = E(5)/D;

Another useful feature of our *KAPattern *program is that it can deal with the irreversible reaction steps. This feature is accessed through the input file. For example, assume that we can neglect the reaction from state 1 to state 2 and from state 2 to state 4. In this case, the input file is simply modified by deleting the lines '1 2 k_6*P' and '2 4 k_5'. The generated link matrix ℒ will be the same as above. But the kinetic matrix  matrix will be modified:

(10)

Correspondingly, the numerator part in the output file will be changed to

%% numerators

E(1) = k6*k_1*k_3*k5*C + k6*k_1*k_3*k_2 + k6*k_1*k_3*k_4 + k6*k_1*k5*C*k4*A + k6*k_1*k_2*k4*A + k6*k_3*k5*C*k2*B + k6*k_3*k_4*k2*B + k6*k5*C*k2*B*k4*A;

E(2) = k5*C*k2*B*k1*A*k_3 + k5*C*k4*A*k3*B*k_1 + k5*C*k2*B*k4*A*k1*A + k5*C*k2*B*k4*A*k3*B;

E(3) = k1*A*k6*k_3*k5*C + k1*A*k_2*k6*k_3 + k1*A*k6*k_3*k_4 + k1*A*k6*k5*C*k4*A + k1*A*k_2*k6*k4*A + k_2*k4*A*k3*B*k6;

E(4) = k2*B*k1*A*k6*k_3 + k4*A*k3*B*k6*k_1 + k2*B*k4*A*k1*A*k6 + k2*B*k4*A*k3*B*k6;

E(5) = k3*B*k6*k_1*k5*C + k3*B*k6*k_1*k_2 + k3*B*k_4*k6*k_1 + k_4*k2*B*k1*A*k6 + k3*B*k6*k5*C*k2*B + k3*B*k_4*k6*k2*B;

## Graphical User Interface (GUI)

**Figure 4:**
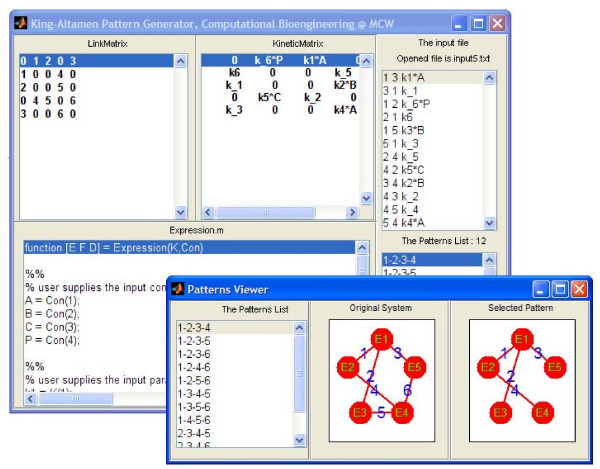
**A screen-shot of the *KAPattern *program GUI for enzyme catalyzed reaction system of fumarase**.

Our stand-alone *KAPattern *package is developed using MATLAB GUI. The executable program is available in Windows, Mac, or Linux formats that do not require MATLAB installed on the end-user's computer. Furthermore, end-users do not need any particular computer programming knowledge to use the package. The GUI has different windows that can display various components of the program, such as input file, link matrix ℒ, kinetic matrix , generated KA pattern list, and MATLAB .*m *output file. There is also a separate window called *Pattern Viewer *which provides users the flexibility to visualize any selected KA pattern, like that shown in Figure 2A. Users can drag and drop nodes and links of one pattern to where they want to deploy them. As an example, a screen-shot of the fumarase enzyme-catalysed reaction system is shown in Figure 4. Users can provide their own input.txt file defining any specific enzyme catalyzed reaction, and run the program in the GUI to view all the valid KA patterns and generate the corresponding rate equation. In addition, the program can generate MathML and MATLAB .m output file in the end-user's working directory. (See the additional file 1: Appendix.)

## Conclusion

We have described a systematic method and the corresponding computer program, called *KAPattern*, for generating rate equations for any complex enzyme systems. This program generates complete set of valid King-Altman patterns for complex enzyme-catalyzed reaction mechanisms. Unlike other computer-assisted methods that use symbolic algebra packages to solve the system of nonlinear algebraic equations arising from steady-state mass conservation, our program is developed from the original schematic method of King-Altman [8] and employs the topological theory of linear graphs [27]. Our program can derive rate equations for both strictly steady-state conditions and those with rapid equilibrium steps. The enzyme mechanism can be either branched or unbranched enzyme mechanisms containing both reversible and irreversible reactions steps. Using a simple, easy-to-understand input file, our program can produce a MATLAB .m file or MathML file that can be integrated into other biochemical system model programs. It can illustrate the visualization of all the valid KA patterns as well. In addition, the generated link matrix ℒ and kinetic matrix , which characterize the enzyme mechanisms here, may be useful for other applications (e.g. to characterize the topological properties and stoichiometric matrix of large-scale networks).

It should be emphasized that in the current version, our program is restricted to systems whose element reactions are association or dissociation of substrates or first-order inter-conversion of enzyme species.

Systems involving allosteric activation and inhibition or other protein-protein interactions should be handled carefully, because our approach still lacks direct connections between the rate constants and the kinetic constants, such as Michaelis-Menten constants. Those connections are important for analyzing enzyme kinetic experimental data.

## Availability and requirements

The *KAPattern *is written in MATLAB and distributed as a standalone GUI-based application for Windows, Mac or Linux/Unix. The MATLAB source codes, and the *KAPattern *stand-alone program are freely available and can be accessed at .

## Authors' contributions

Basic idea was conceived by FQ. Software was developed and implemented by FQ and YH, with RKD and DAB advising. The manuscript was written by FQ and revised by RKD and DAB. Manuscript was read and approved by all authors.

## Supplementary Material

Additional file 1 **Appendix for "Generating rate equations for complex enzyme systems by a computer-assisted systematic method"**. Contains the brief description of all of the functions in the package and a full example to demonstrate the use of the package. Click here for file
