# Supplementary material for: Generating rate equations for complex enzyme systems by a computer-assisted systematic method
Source: BMC Bioinformatics. 2009 Aug 4;10:238. doi: 10.1186/1471-2105-10-238 (PMC2729780; doi:10.1186/1471-2105-10-238)
Supplement: Additional file 1 — Appendix for "Generating rate equations for complex enzyme systems by a computer-assisted systematic method". Contains the brief description of all of the functions in the package and a full example to demonstrate the use of the package. [file 1471-2105-10-238-S1.pdf]

Appendix for "Generating Rate Equations for Complex Enzyme Systems by a Simple Computer-Assisted Systematic Method"

Feng Qi, Ranjan K. Dash, Yu Han and Daniel A. Beard

Biotechnology and Bioengineering Center and Department of Physiology, Medical College of Wisconsin, Milwaukee, Wisconsin.

http://www.biocoda.org

1. Using the Stand-Alone Package

The stand-alone package (available for Windows, Mac, or Linux) can be run using the executable KApattern.exe (Windows), sh run_KA.sh (Mac), or run_KA.sh (Linux). Executing the package, a graphical interface will appear on the user's screen:

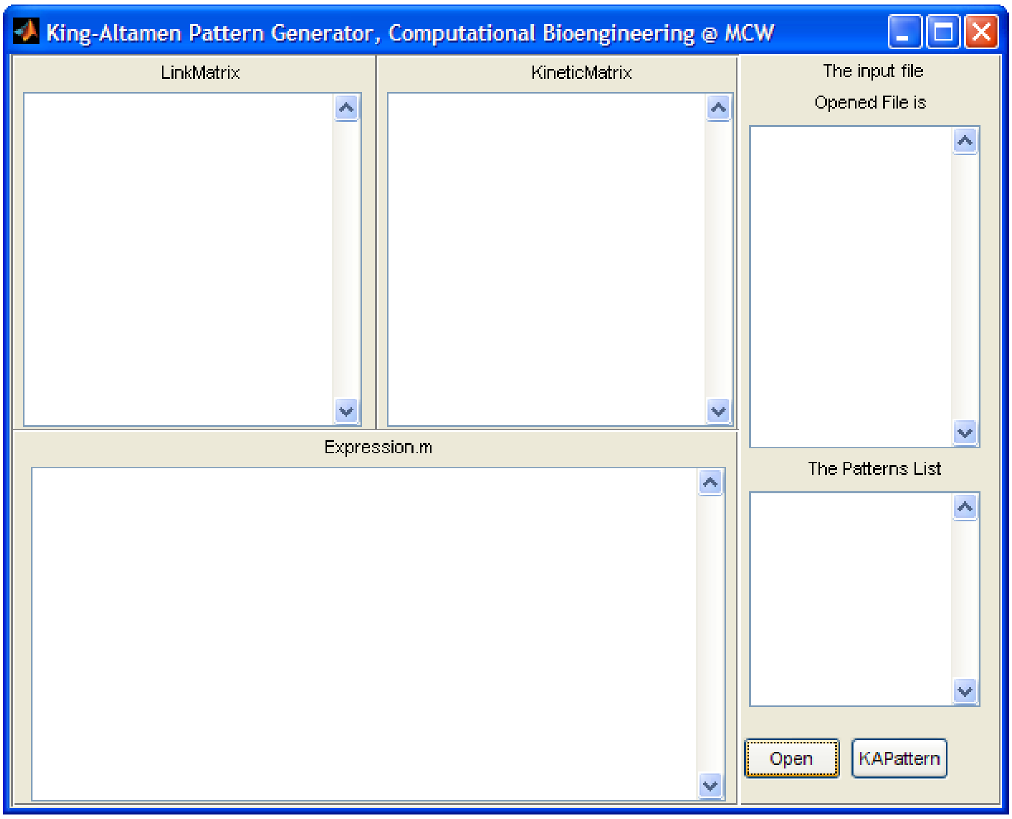

The first thing necessary to use the package is an input file. Two example input files are included in the distribution. For the fumarase example presented in the main body of the paper, the input file is

```
fumarase.txt:
1 3 k1*A
3 1 k_1
1 2 k_6*P
2 1 k6
1 5 k3*B
5 1 k_3
2 4 k_5
4 2 k5*C
```

3 4 k2*B
4 3 k_2
4 5 k_4
5 4 k4*A

The input file is loaded by clicking the button “open” on the interface and selecting the desired input file.

Next, upon clicking the button “KAPattern” the program will generate all valid patterns and display the results. The link and kinetic matrices and the equations for the flux expression are presented in the main window:

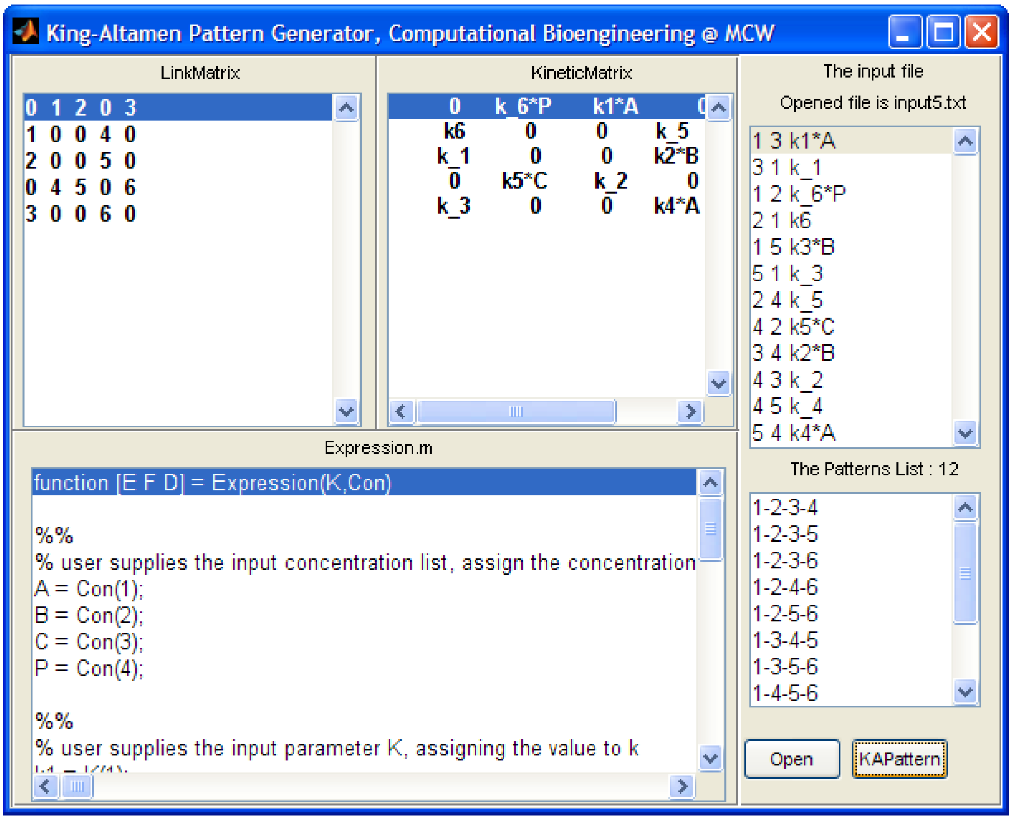

and the KA patterns are visualized in a separate window called “Patterns Viewer”:

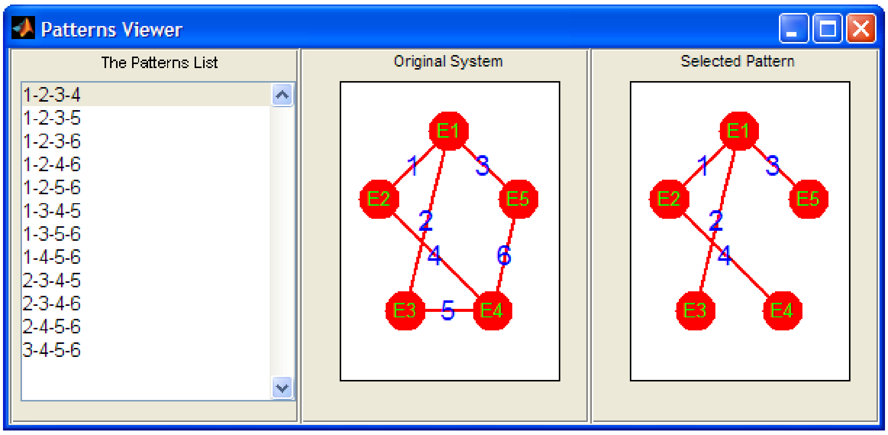

Users can select different patterns to visualize from the list on the left, and can drag-and-drop nodes to different positions in the diagram.

In addition to the graphical output, output files `Expression.m` and `ResultPage.html` are generated and saved in working directory. The files `Expression.m` lists the equations associated with the flux expression in MATLAB syntax. The file `ResultPage.html` lists the flux expression in MathML syntax.

2. Moderate-Sized Example

Here we consider a moderate size system, the random bi-bi reaction with no dead-end complexes (Segel 1993, p307), as shown below.

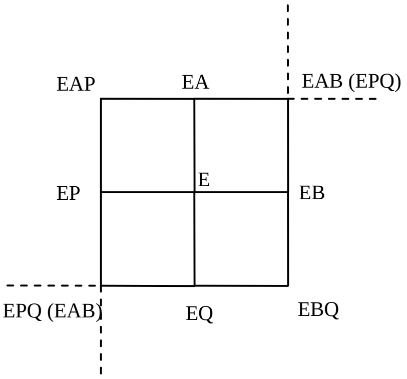

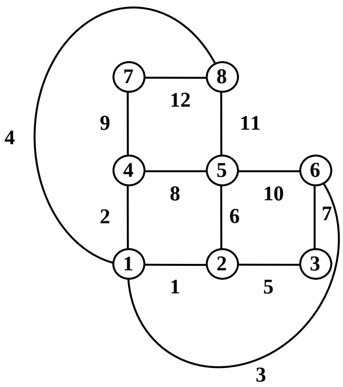

Two graphic representations of this mechanism are shown above. In totally, there are eight enzyme forms and twelve links. The input file for this system is:

```
bi-bi.txt:
1 4 k1
4 1 k_1
1 2 k2
2 1 k_2
2 3 k3
3 2 k_3
3 6 k4
6 3 k_4
2 5 k5
5 2 k_5
4 5 k6
5 4 k_6
4 7 k7
7 4 k_7
7 8 k8
8 7 k_8
5 8 k9
8 5 k_9
5 6 k10
6 5 k_10
1 8 k11
8 1 k_11
1 6 k12
6 1 k_12
```

(This example input file is distributed with the package.) For simplicity, all rate constants are assumed pseudo-first-order rate constants here.

Upon loading the input file, the program will generate the *KineticMatrix* and the *LinkMatrix*:

$$L = \begin{pmatrix} 0 & 1 & 0 & 2 & 0 & 3 & 0 & 4 \\ 1 & 0 & 5 & 0 & 6 & 0 & 0 & 0 \\ 0 & 5 & 0 & 0 & 0 & 7 & 0 & 0 \\ 2 & 0 & 0 & 0 & 8 & 0 & 9 & 0 \\ 0 & 6 & 0 & 8 & 0 & 10 & 0 & 11 \\ 3 & 0 & 7 & 0 & 10 & 0 & 0 & 0 \\ 0 & 0 & 0 & 9 & 0 & 0 & 0 & 12 \\ 4 & 0 & 0 & 0 & 11 & 0 & 12 & 0 \end{pmatrix}$$

$$K = \begin{pmatrix} 0 & k_2 & 0 & k_1 & 0 & k_{12} & 0 & k_{11} \\ k_{-2} & 0 & k_3 & 0 & k_5 & 0 & 0 & 0 \\ 0 & k_{-3} & 0 & 0 & 0 & k_4 & 0 & 0 \\ k_{-1} & 0 & 0 & 0 & k_6 & 0 & k_7 & 0 \\ 0 & k_{-5} & 0 & k_{-6} & 0 & k_{10} & 0 & k_9 \\ k_{-12} & 0 & k_{-4} & 0 & k_{-10} & 0 & 0 & 0 \\ 0 & 0 & 0 & k_{-7} & 0 & 0 & 0 & k_8 \\ k_{-11} & 0 & 0 & 0 & k_{-9} & 0 & k_{-8} & 0 \end{pmatrix}$$

In addition, 288 valid KA patterns will be generated.

3. The MATLAB Package and Source Code

The MATLAB package can be run much the same way as the stand-alone package. The command to launch the GUI interface is `Main.m`.

The table below list the functions included in the MATLAB source code.

**Function Name**	**Description**
GUI functions	
`Main.m`	The main drive script of GUI for the KAPattern
`SubMain.m`	Program used to generate the sub-windows for displaying the valid patterns.
`MyGUIEdit.m`	Called by `SubMain.m`, function used to perform the drag-drop feature to let users customize the layout of the KA-patterns in sub-windows.
Functions implementing the algorithm	
`KAPattern.m`	Main function for KAPattern package, called by Main.m
`ReadInput.m`	Function is used to read the input file of enzyme catalytic reaction system and get the necessary information from the input file.
`GetLink.m`	Function is used to get *LinkMatrix* and *LinkList*.
`Wang.m`	Function is used to perform the Wang Algebra alphanumerically multiplication.
`GetExpression.m`	Function is used to get expressions for each enzyme

	form given valid K-A patterns
`BuildFile.m`	Function is used to build the output the .m and the MathML files after get the expressions.

For detailed help on each function, type ‘`help FunctionName`’ in MATLAB commend line.

The `ReadInput` and `GetLink` functions are used to read the input file and analysis the input system, and then generate necessary parameters, such as *KineticMatrix*, *LinkMatrix*, and *LinkList*, which are needed to compute valid KA patterns for the input system. Detailed explanations of the usage (input/output) and the meaning of each function can be obtained from the functions’ help pages. The indexes of each link in the system are assigned automatically by the function `GetLink` and the result is saved in *LinkMatrix*.

The `KAPattern` is the main function for the package if users do not want to use GUI feature. The function will give four output parameters. They are expression corresponding to each enzyme form derived from valid KA patterns, the denominator expression of enzyme fractions, the rate constants list, and the substrates or products concentration lists which show up in the pseudo-first-order rate constants respectively.

References

Segel, I. H. (1993). “Enzyme kinetics.” *John Wiley & Sons, Inc.*
